# The Impact of Significant Geographical Barriers on the Invasion Risk of Non-Native Aquatic Animals: A Case Study of the Qinling Mountains, China

**DOI:** 10.3390/biology15040329

**Published:** 2026-02-13

**Authors:** Xin Wang, Chen Tian, Xiaoyu Jia, Yahui Zhao, Yingchun Xing

**Affiliations:** 1 Resource and Environmental Research Center, Chinese Academy of Fishery Sciences, Beijing 100141, China; xin37209@gmail.com; 2 State Key Laboratory of Animal Biodiversity Conservation and Integrated Pest Management, Institute of Zoology, Chinese Academy of Sciences, Beijing 100101, China; tianc.qd@foxmail.com; 3 National Demonstration Center for Experimental Fisheries Science Education, Shanghai Ocean University, Shanghai 201306, China; 4 Shanxi Provincial Aquatic Technology Extension Service Center, Xiaodian District, Taiyuan 030006, China; sxtech@163.com

**Keywords:** species distribution models, high-invasion-risk, invasion risk, human activities

## Abstract

Biological invasion is a major driver of biodiversity loss and ecosystem disruption, with non-native aquatic species threatening ecological integrity and economic stability. The Qinling Mountains, located in central China, serve as a crucial barrier between temperate and subtropical climate zones, and separate the Yellow and Yangtze River basins. This study investigates the role of these geographical barriers in regulating the distribution and invasion risk of non-native aquatic species. We identified 27 non-native species in Shaanxi Province based on occurrence records compiled from field survey conducted between 2012 and 2024 (and from 2019 to 2024 in the Yellow River mainstream of the Shanxi–Shaanxi Gorge), including 13 high-risk species, such as *Trachemys scripta elegans*, *Procambarus clarkii*, *Sander lucioperca*, and *Hypomesus olidus*. Using the Aquatic Species Invasiveness Screening Kit and species distribution models, we identified the Hanjiang River in the Yangtze basin and Weihe River estuary in the Yellow River basin as high-risk areas for these species. Mean annual temperature was the primary environmental factor influencing species distribution, with species adapted to cooler conditions predominantly found north of the Qinling Mountains, while those preferring warmer climates are more common in the south. Our findings highlight the Qinling Mountains as both a physical and climatic barrier, limiting cross-basin dispersal and creating distinct invasion patterns. However, human activities such as inter-basin water-transfer projects, damming, and aquaculture practices have gradually weakened the barrier’s effectiveness, facilitating the spread of invasive species. We recommend prioritizing monitoring efforts in cross-basin water-transfer regions, focusing on high-risk species adapted to both cooler and warmer climates, and incorporating environmental DNA (eDNA)-based monitoring in recipient areas of inter-basin water-transfer projects for early detection and control to minimize ecosystem damage.

## Simple Summary

Biological invasions are an important cause of biodiversity loss. The Qinling Mountains form a key natural barrier in central China: they separate the warmer south from the colder north, and they also lie between the Yangtze and Yellow River basins. Using field surveys and existing distribution records, we examined whether this barrier affects the invasion risk of non-native aquatic animals and whether their distributions differ on the two sides of the mountains. Our results show that temperature is the strongest factor shaping invasion risk. Species that tolerate colder conditions are more common north of the Qinling Mountains, while species that prefer warmer conditions are more common to the south. However, human activities—such as water-transfer projects, dam building, and aquaculture—can reduce the barrier effect by creating new pathways for movement between river basins. We therefore suggest giving priority to monitoring areas where water systems are connected by engineering projects and strengthening early detection and rapid action to reduce potential ecological harm.

## 1. Introduction

Biological invasions are a leading driver of biodiversity loss and ecosystem disruption, with significant ecological, economic, and societal impacts worldwide [1,2]. Non-native aquatic species pose particularly severe threats, often outcompeting native species, destabilizing ecosystems, and facilitating the spread of diseases [3]. Human-mediated activities, such as aquaculture, shipping, and recreational fishing, exacerbate these invasions, frequently overcoming natural dispersal barriers [4,5].

Geographical features, including elevation and topography, play a critical role in shaping environmental conditions, influencing key factors, such as temperature, moisture, and light availability, which in turn affect species establishment and spread [6,7]. In terrestrial ecosystems, climate variables like temperature and precipitation directly impact organismal survival, while topographic features such as slope and aspect create microclimatic variations that indirectly shape species distributions [8,9]. These dynamics extend to aquatic ecosystems, where they similarly determine the success of non-native species invasions [10,11].

China, as a global hotspot for non-native aquatic species introductions, exemplifies the challenges posed by biological invasions [12,13]. Rapid growth in the aquaculture and ornamental trade industries has driven the introduction of at least 439 of the 624 globally recorded non-native freshwater fish species into China, making it one of the countries with the highest number of introduced fish species [14]. Notable examples such as Oreochromis niloticus, Sander lucioperca, and Procambarus clarkii have established invasive populations with significant ecological and economic consequences [15,16,17]. The patterns of invasion and species composition vary significantly between southern and northern China, reflecting the influence of vast geographical diversity, including five distinct climatic zones and complex natural barriers [18]. Among these barriers, the Qinling Mountains serve as a pivotal natural divide, separating subtropical and temperate regions and regulating the dispersal of non-native species.

The Qinling Mountains hold an extremely important position in China’s physical geography, serving as the boundary between the northern and southern regions of the country [19,20]. The Qinling Mountains not only lie between the subtropical and warm temperate zones, but also act as a significant watershed between the Yellow River and Yangtze River systems in China [21]. North of the Qinling Mountains lies the Yellow River basin, which is primarily traversed by the Weihe River and Luohe River. To the south, the Yangtze River basin extends, featuring significant rivers like the Hanjiang and Jialingjiang Rivers [19]. Notably, the Weihe River stands as the largest tributary of the Yellow River, and the Hanjiang River is the longest tributary of the Yangtze River [22]. The Qinling Mountains act as a natural divide between northern and southern China, effectively impeding the passage of moist maritime air masses into the northwest and obstructing the incursion of cold northern air into the southern regions [23,24]. This geographical feature significantly contributes to the temperature disparities observed between these two areas. The Qinling Mountains (in the narrower sense) are confined to the southern mountainous region of Shaanxi Province, bounded by the Weihe River to the north and the Hanjiang River to the south, with the Bahe River and Danjiang River basins to the east and the Jialingjiang River to the west [22,25]. Additionally, influenced by the geological structure of the Qinling Mountains, the northern slopes are steep, with short, fast-flowing rivers, whereas the southern slopes are gentler, with longer, slower-flowing rivers [26]. This diversity in terrain and hydrology creates distinct environmental conditions on either side of the mountains, shaping the establishment and spread of aquatic species. Furthermore, the Qinling Mountains serve as a crucial biogeographic boundary in global animal geography, delineating the transition zone between the Palearctic and Oriental regions [27]. This divide influences not only climate patterns and water systems but also plays a pivotal role in the distribution and diversification of species across China [26].

Despite their recognized importance, the role of geographical barriers in regulating aquatic invasions, particularly in regions with distinct climatic gradients and complex hydrological networks, remains insufficiently quantified. Previous studies have shown that climatic regimes and basin boundaries are important factors limiting the establishment and spread of non-native aquatic species [28,29]. Most studies rely on qualitative descriptions, with limited integration of predictive modeling approaches. To address this gap, we focus on the Qinling Mountains, a significant geographical barrier in China, to evaluate its influence on the invasion risk of high-risk non-native aquatic animals across contrasting climatic zones. By combining species distribution models, random forest analysis, and geographical barriers as spatial filters, we analyze the potential distribution patterns of these species and examine how environmental factors associated with this natural barrier shape their dispersal and establishment. This study not only quantifies the extent to which the Qinling Mountains constrain species dispersal and invasion risk but also provides a robust framework for understanding how natural barriers regulate the spread of invasive species in heterogeneous landscapes. The findings will offer valuable insights to inform strategies for preventing further invasions and protecting aquatic biodiversity.

## 2. Materials and Methods

### 2.1. Collection of Species Distribution Information

**Figure 1:**
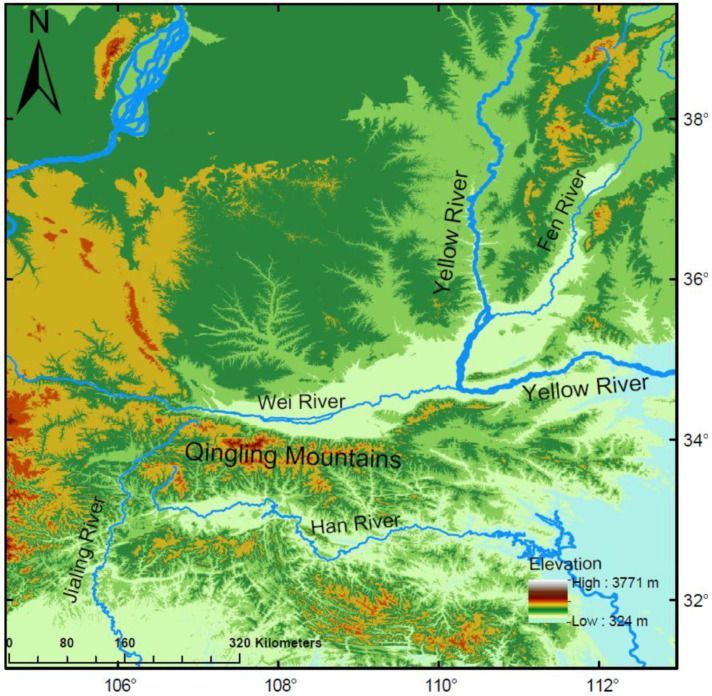
Geomorphological map of the Qinling Mountains.

This study involves aquatic animals, primarily including fish, reptiles, and crustaceans. The data sources are divided into two parts. First, we compiled the non-native aquatic species inventory for the study area. The inventory data were primarily sourced from field surveys conducted between 2012 and 2024 in the Weihe River basin on the northern slope of the Qinling Mountains, the Hanjiang River basin on the southern slope, and from 2019 to 2024 in the Yellow River mainstream of the Shanxi–Shaanxi Gorge (Figure 1).

Additionally, historical literature records and specimen records collected from the Qinling Mountains since 1930, stored in the National Animal Collection Resource Center, Institute of Zoology, Chinese Academy of Sciences, Beijing, China (ASIZB), were also included. For the purpose of predicting the potential suitable distribution areas of non-native aquatic species, we used a broader set of species distribution data. These data, which cover a larger geographic range than the study area, were sourced from multiple platforms, including the Global Biodiversity Information Facility (GBIF) website (https://www.gbif.org/, accessed on 13 January 2024) and the World Fish Database (FishBase, https://www.fishbase.org/, accessed on 16 March 2024) [30,31], as well as historical literature records and field survey data from this study. These sources provided a more extensive view of species distribution, which was crucial for making accurate predictions across a wider region. After data integration, all collected species distribution data were rigorously screened and corrected to ensure accuracy for subsequent analyses. Specifically, we checked all occurrence records for duplicate entries, including identical geographic coordinates and repeated records from the same source. Because the target taxa are aquatic animals, we further evaluated the positional accuracy of each record based on the global river network dataset (https://www.hydrosheds.org/, accessed on 10 June 2024) at 30 s resolution. Using ArcGIS Desktop 10.8 (Esri, Redlands, CA, USA; accessed on 21 January 2024), we corrected the spatial position of occurrence points to ensure that all records were located within or adjacent to natural water bodies. Specifically, records falling outside mapped water bodies were manually checked and, when appropriate, snapped to the nearest river/lake feature so that the final coordinates were positioned within or immediately adjacent to the water network. This process minimized errors and provided a reliable foundation for predicting potential suitable areas for species.

Species introduction information is mainly divided into several aspects. (1) Sources of introduction: abroad (AB); Other regions of China (ORC); Different sections of the same river basin (DSB). (2) Pathways of introduction: Active (Aquaculture, Stocking for Fisheries, Ornamental Trade), and Passive (Unintentional transport).

### 2.2. Non-Native Aquatic Species Classification Criteria

In this study, we used a multi-tiered approach to determine whether species were classified as non-native aquatic animals. First, we referred to the International Union for Conservation of Nature (IUCN) Red List of Threatened Species (https://www.iucn.org/, accessed on 3 March 2024) to obtain species distribution data as the initial screening criterion. Since the IUCN data have relatively coarse resolution, we further incorporated regional sources, including the List of Invasive Alien Species in China’s Natural Ecosystems (https://www.mee.gov.cn/gkml/hbb/bgg/201612/t20161226_373636.htm, accessed on 14 March 2024), Species diversity and Distribution of Inland Fishes in China, Fishes of the Qinling Mountains, and Fishes of the Yellow River, to refine the classification of native and non-native species. To further verify species identity and distribution status, we conducted expert consultations with regional ichthyologists and taxonomists to cross-check ambiguous or conflicting records according to the predefined criteria; these consultations served only as supporting validation and were not used as the sole basis for classification. In cases where data from different sources were inconsistent, we made comprehensive judgments based on the timeliness of the data, authority, and applicability. Based on this process, we ultimately compiled a complete list of non-native aquatic animals in the study area.

### 2.3. Environmental Variable

The environmental data are from the BCC-CSM2-MR model of the sixth Coupled Model Intercomparison Project (CMIP6), selecting the Shared Socioeconomic Pathways (SSPs) with a moderate development scenario (SSP245) for the period of 2021–2040. This time includes 19 environmental climate variables and 1 topographic variable (Table 1), with the environmental climate variables mainly being temperature and precipitation. All environmental predictors have been previously used in modeling studies of other freshwater invasive species [32]. In addition, these data were used at a spatial resolution of 2.5 arc-minutes.

### 2.4. Toolkit Description

Risk screenings were undertaken using the Aquatic Species Invasiveness Screening Kit V2.3 (AS-ISK) [33]. During the assessment, the following two principles should be considered: (1) Whether the species has a history of invasion, distinguished by the numbers 1 and 0, with 1 indicating that the species has a history of invasion, and 0 indicating that the species does not have a history of invasion. (2) The ability of the species to reproduce naturally and form a stable population. The tool consists of 55 questions, with the first 49 questions for basic risk assessment and the last 6 questions for climate change risk assessment. Within AS-ISK, questions 1–49 constitute the Basic Risk Assessment (BRA), which evaluates invasion risk along the introduction, establishment/spread, and potential impact pathway. Questions 50–55 comprise the Climate Change Assessment (CCA) and examine how projected climatic conditions may modify these risks (i.e., risks related to introduction, establishment, dispersal, and impact under changing climatic scenarios), yielding a combined BRA + CCA score. Additionally, when assessing the questions, the basis for each question and the corresponding level of confidence must be provided (four levels of confidence: low = 1, medium = 2, high = 3, very high = 4). After the assessment, the total score was calculated based on the scores of the corresponding questions.

This study used the Receiver Operating Characteristic (ROC) curve to determine the accuracy of the assessment results [34,35]. The ROC curve was plotted using SPSS Statistics 26.0 (IBM Corp., Armonk, NY, USA). The horizontal axis of the ROC curve represents specificity, and the vertical axis represents sensitivity. The area under the curve ranges from 0.5 to 1, with a closer value to 1 indicating higher accuracy of the assessment results [36]. The Youden’s index was calculated using the results of the ROC curve, and then the assessment risk threshold was determined based on the Youden’s index results. The threshold is mainly used to distinguish whether the species are high-invasion-risk or middle-invasion-risk species [37].

### 2.5. Ecological Niche Modeling

Based on the results of the Aquatic Species Invasiveness Screening, potential suitable distribution areas were predicted for high-invasion-risk non-native aquatic animals using the MaxEnt model [38]. To ensure reliable occurrence data and reduce potential sampling bias and spatial autocorrelation, the occurrence dataset was spatially filtered prior to modeling, which also minimizes the risk of pseudo-absence. Species distribution points were cleaned using ArcGIS and Excel software. MaxEnt, a presence-only modeling approach, accounts for pseudo-absence by using background points sampled across the study area. Environmental data and species occurrence records were input into the model, with 75% of the data allocated for training and 25% for testing. This split follows a commonly used MaxEnt holdout setting (random test percentage = 25) to retain an independent subset for model evaluation [39]. Model performance was evaluated using the Area Under the Curve (AUC) of the Receiver Operating Characteristic, where AUC values above 0.7 indicate good predictive ability. Although ensemble models using multiple algorithms can enhance robustness, MaxEnt has been widely applied in species distribution modeling, including for aquatic animals, and has consistently shown high predictive performance for presence-only data [38,40]. Finally, predicted distribution areas were combined with geographic base map data from the 1:4 million national basic geographic dataset to generate the potential suitable distribution maps for high-invasion-risk aquatic animals.

### 2.6. High-Invasion-Risk Non-Native Aquatic Animals-Environmental Factors Relations

Environmental data were extracted from the WorldClim website (https://www.worldclim.org, accessed on 14 November 2024) for environmental and topographic factors [41], and environmental data for distribution areas were obtained through species presence data. Due to the multicollinearity among environmental variables, to ensure accurate environmental data for species distribution and to avoid fitting phenomena between data, it was necessary to select environmental variables [42]. The selection process involves saving the files as txt file format and importing them into SPSS software for bivariate correlation analysis. When the correlation |r| > 0.8, variables were retained based on the following two requirements: (1) Variables that had significant meaning for species distribution; (2) Based on the contribution rate of environmental factors. Selection was made according to these two principles. Dominance analysis was conducted using redundancy analysis (RDA), a multivariate method that explores relationships between species composition and environmental variables. In this study, the RDA used the selected environmental variables as explanatory variables and species presence/absence matrices as response variables, where “presence” indicates the species was observed in surveys or recorded historically, and “absence” indicates it was not detected during surveys. RDA combines species and environmental data through linear models to maximize the correlation between species distribution and environmental factors. The relative contribution of each environmental variable was evaluated based on the direction and length of its vector in the RDA biplot, with longer vectors indicating stronger influence on species distribution [43,44,45].

### 2.7. Drivers of High-Invasion-Risk Non-Native Aquatic Animals

The Random Forest (RF) model was used to establish a linear relationship between environmental factors and the distribution of high-invasion-risk non-native aquatic animals. RF is an algorithm based on classification and regression trees as meta-classifiers, referring to a widely used machine learning model that combines bagging and random features [46]. The model works by using the training dataset containing both the response and predictor variables, associated distance matrix, and distance thresholds [47]. It generates multiple classification trees, and outputs the results using voting or arithmetic averaging methods based on the results of all classification trees. At the same time, it uses the Shapley Additive exPlanations (SHAP) index for variable importance ranking to describe the contribution rate of feature variables to the results, providing a reasonable explanation [48]. The Random Forest model can smoothly handle overfitting issues between data when dealing with large datasets, and can accurately assess feature variables that affect the final results from a large number of feature variables [49,50]. By selecting the results of redundancy analysis, environmental variables of significant importance to species are included in the Random Forest model.

## 3. Result

### 3.1. Current Status of Non-Native Aquatic Animals in the Qinling Mountains

A total of 27 non-native aquatic species were identified, belonging to 2 phyla, 4 classes, 9 orders, and 16 families. Among these, fish represent the largest group, comprising 21 species across 7 orders and 10 families. Additionally, there are 2 crustacean species from 1 order and 2 families, 3 reptile species from 1 order and 3 families, and 1 amphibian species from 1 order and 1 family. Of these, 12 species/varieties were introduced from abroad (AB), 14 species/varieties were introduced from other regions of China (ORC), and 1 species/varieties were introduced from different sections of the same river basin (DSB). A breakdown of the introduction pathways shows that non-native aquatic animals in the tributaries of the Yangtze River basin (YZRB) in Shaanxi were introduced via aquaculture (8 species), Unintentional transport (1 species), Stocking for Fisheries (1 species), and Ornamental Trade (1 species). In the Yellow River basin (YRB) within Shaanxi, the non-native species were introduced through aquaculture (18 species), Unintentional transport (1 species), Stocking for Fisheries (2 species), and Ornamental Trade (4 species) (Table 2).

Additionally, statistical results show differences in species distribution between the tributaries of the YZRB and the main and tributary streams of the YRB in Shaanxi. Specifically, the Hanjiang River, the primary tributary of the YZRB, hosts 10 non-native aquatic species, while the Jialingjiang River has none. In the main stream of the YRB, 23 non-native species were recorded, and 5 species were identified in the Weihe River (Table 3).

### 3.2. Invasion Risk Assessment

Based on the Aquatic Species Invasiveness Screening Kit (AS-ISK) results (Table 4), assessment outcomes were divided into Basic Risk Assessment (BRA) scores and composite scores (BRA + CCA). According to the Youden index threshold calculated using SPSS software, a score of 32.75 (composite score) is the cutoff. Species with a composite score ≥ 32.75 and a documented invasion history (IS) are classified as high invasion risk. Species with a composite score greater than 1 but ≤ 32.75, and with an IS, are considered medium invasion risk. Species with a composite score less than 1 are classified as low or no invasion risk.

In total, 13 non-native aquatic species were identified as high-risk, 5 as medium-risk, and 9 as low or no invasion risk. Overall, most high-invasion-risk non-native aquatic animals showed consistently high values under both BRA and composite (BRA + CCA) scores, indicating that their invasion potential remains high even without considering climate change. However, several species exhibited moderate BRA scores but were elevated to a higher risk category after the inclusion of climate change factors (CCA), suggesting that future climatic shifts may further increase their invasion potential.

### 3.3. Predicted Suitable Habitats for High-Invasion-Risk Non-Native Aquatic Animals

**Figure 2:**
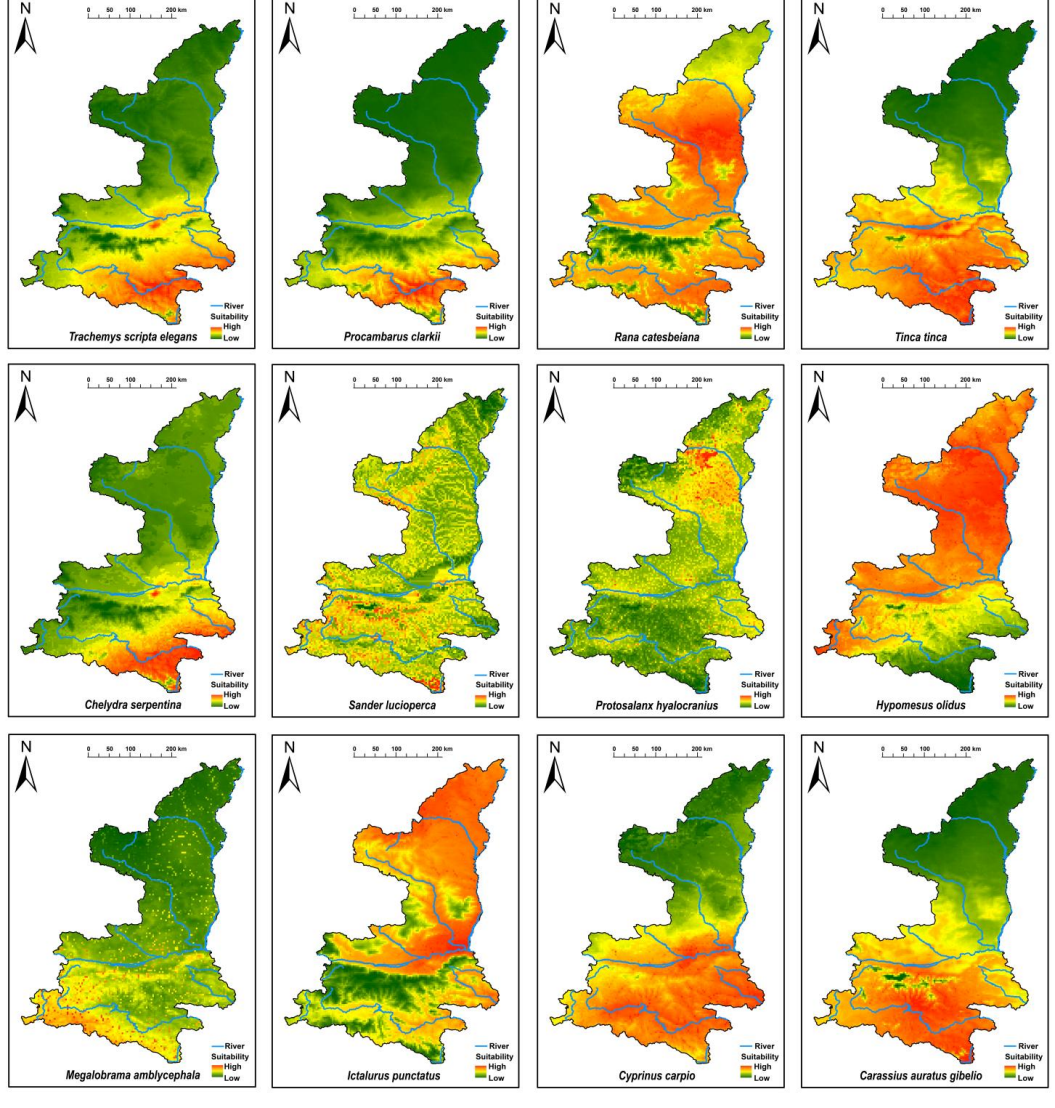
Prediction results of potential suitable region for non-native aquatic animals with a high invasion risk in Shaanxi Province. Note: Map was created using the administrative boundaries of Shaanxi Province for reference.

Based on the AS-ISK assessment results, a potential habitat suitability prediction analysis was conducted for high-invasion-risk non-native aquatic animals in Qinling Mountains using the Maxent model, (model performance metrics and suitability-class thresholds are provided in Table S1), as shown in Figure 2. The predicted suitable habitats for species/variety such as *Sander lucioperca*, *Protosalanx hyalocranius*, *Hypomesus olidus*, *Ictalurus punctatus*, *Cyprinus carpio*, and *Rana catesbeiana* cover relatively large areas. In contrast, species/variety like *Trachemys scripta elegans*, *Procambarus clarkii*, and *Megalobrama amblycephala* have smaller predicted suitable areas.

In terms of the regions on both sides of the Qinling Mountains, the invasion areas of most high-invasion-risk non-native aquatic animals are concentrated in the southern regions of the Qinling Mountains. However, a few high-invasion-risk non-native aquatic animals, such as *Protosalanx hyalocranius* and *Hypomesus olidus*, have their invasion areas primarily located in the northern regions of the Qinling Mountains (Figure 2).

Four rivers present the highest invasion risks for non-native aquatic species, with two in the YRB (Weihe River and Jinghe River) and two in the YZRB (Hanjiang River and Jialingjiang River). These river habitats are highly suitable for the invasion of most non-native animals. In contrast, compared to the rivers in southern Shaanxi, the rivers in the central region, such as the Wudinghe River, show relatively lower invasion risks.

### 3.4. Ordination of Environmental Factor Assemblies

**Figure 3:**
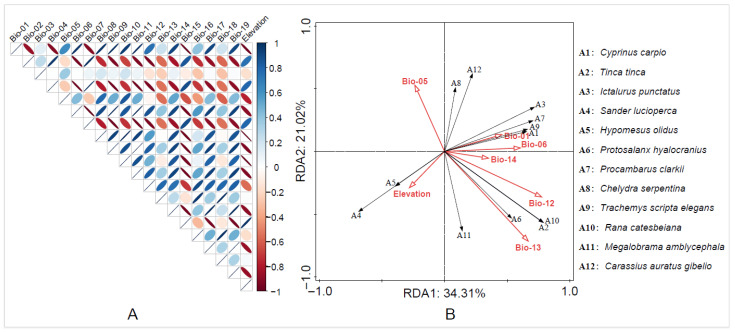
(**A**) Principal component analysis (PCA) results based on environmental variables; (**B**) redundancy analysis (RDA) results for high-invasion-risk non-native aquatic animals. Note: Species abbreviations A1–A12 correspond to the twelve focal non-native aquatic animal species and are used as codes (“A” + number) in the figure.

Considering the impact of extreme climate thresholds on species distribution and dispersal, seven environmental variables (Bio-01, Bio-04, Bio-05, Bio-12, Bio-13, Bio-14, and Elevation) were retained based on the Pearson correlation results (Figure 3A). These retained environmental variables were used in a redundancy analysis (RDA) (Table S2), which explained 55.75% of the distribution patterns of high-invasion-risk non-native aquatic animals based on presence data and environmental variables. The results show that environmental variables related to temperature are the main variables affecting species distribution.

The RDA1 axis was primarily aligned with environmental factors such as Bio-01 (Annual Mean Temperature), Bio-06 (Min Temperature of Coldest Month), and Elevation. The RDA2 axis is mainly explained by Bio-12 (Annual Precipitation) and Bio-13 (Precipitation of Wettest Month). In terms of species distribution, different species respond to environmental variables in distinct ways. For example, *Cyprinus carpio* shows a correlation with Bio-01 along the RDA1 axis, while *Rana catesbeiana* is associated with Bio-12 and Bio-13 on the RDA2 axis. Additionally, elevation, as a topographic variable, also showed clear alignment with the distribution patterns of multiple species (Figure 3B).

### 3.5. Response Degree of High-Risk Non-Native Aquatic Animals to Environmental Variables

Based on the RDA results, Bio-01 was identified as the primary influencing factor and incorporated into the random forest model. The random forest analysis revealed species-specific responses to Bio-01. Most species exhibited higher suitability within the 0–20 °C range, indicating a significant influence of Bio-01 on species distribution in this temperature range. For instance, *Carassius auratus gibelio*, *Cyprinus carpio*, *Sander lucioperca*, and *Tinca tinca* showed peak responses in the range of 5–20 °C, suggesting adaptability to moderate Bio-01 conditions.

**Figure 4:**
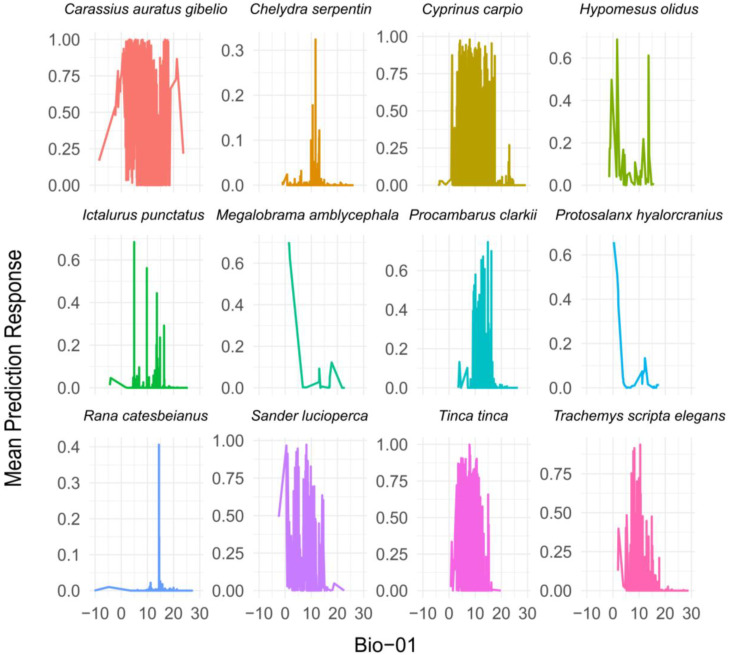
Result of response curves of high-risk non-native aquatic animals to Bio-01 based on random forest.

Species such as *Chelydra serpentina* and *Megalobrama amblycephala* responded strongly within the 0–5 °C range, indicating a preference for colder environments. In contrast, *Rana catesbeiana* and *Hypomesus olidus* exhibited narrower response ranges, reflecting stricter environmental requirements for Bio-01. Beyond 20 °C, the predicted suitability for most species declined sharply, suggesting that elevated Bio-01 values have minimal positive effects on species adaptability, and higher temperatures may limit the survival of most species (Figure 4).

## 4. Discussion

### 4.1. Distribution Patterns of Non-Native Aquatic Species in the Qinling Mountains Region

**Figure 5:**
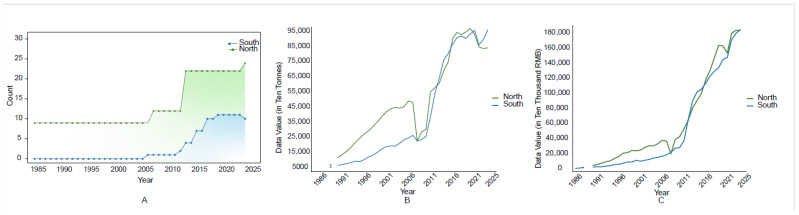
Changes in the increase in non-native aquatic species on the northern and southern sides of the Qinling Mountains (**A**). Changes in production on the northern and southern sides of the Qinling Mountains (**B**). Changes in fishery production value on the northern and southern sides of the Qinling Mountains (**C**). Note: As there are no records of production values for 1986, 1987, and 1989 in (**B**), as well as for 1989 in (**C**), these data points are not displayed in the figures.

The Qinling Mountains, as a natural geographical barrier within Shaanxi Province, play a crucial role in influencing the distribution patterns of non-native aquatic species, particularly in terms of differences in species richness between the northern and southern regions (Figure 5A). In northern Shaanxi, the relatively flat terrain and favorable conditions for large-scale aquaculture have contributed to the rapid development of the aquaculture industry, characterized by significant production value and scale (Figure 5B) [62]. The growth of economic activities, particularly those related to water resource utilization and fisheries development, has created favorable conditions for the introduction of non-native species (Table S3). Studies have shown that the rapid expansion of aquaculture in northern Shaanxi is, to some extent, dependent on the farming of non-native species, further facilitating their introduction and spread [12,63].

In contrast, aquaculture in southern Shaanxi began later and historically involved a limited introduction of non-native species, resulting in relatively lower aquaculture production value. However, recent years have seen rapid development in the aquaculture industry in southern Shaanxi, particularly an increase in fisheries output, which has significantly elevated the opportunities for non-native species introduction (Figure 5C) [54,62]. Despite the natural geographical isolation provided by the Qinling Mountains, the expansion of aquaculture and increasing fisheries activities across Shaanxi Province have gradually narrowed the gap in non-native species introductions between the northern and southern regions, thereby increasing the risk of species spread. This change highlights that, although the Qinling Mountains have historically limited the north–south spread of species within Shaanxi Province, the rapid development of aquaculture and cross-regional trade activities are gradually eroding this barrier, potentially enabling the invasion of more non-native species.

### 4.2. Analysis of the Causes of Non-Native Aquatic Animals’ Introduction

**Figure 6:**
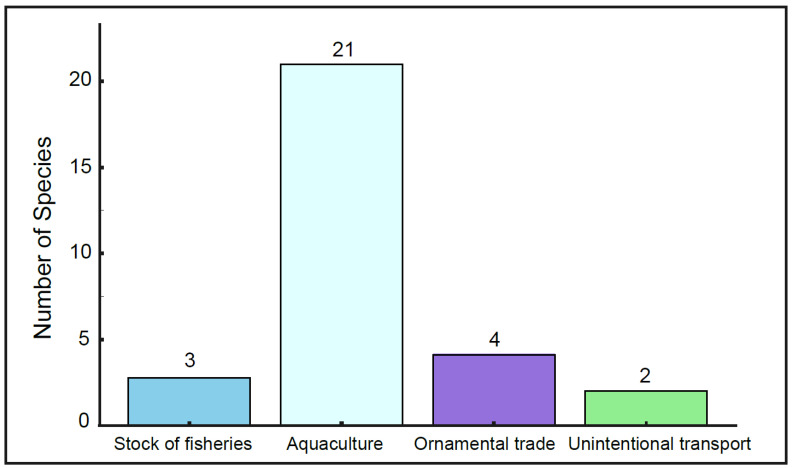
Pathways for the introduction of non-native aquatic animals.

Based on the distribution status of non-native aquatic animals in Qinling Mountains, among introduction ways, aquaculture accounts for the highest number of species, with 21 species, followed by the ornamental trade, with 4 species; stock of fisheries, with 3 species; and unintentional transport, with 2 species (Figure 6). Aquaculture introductions are the most important pathway for introducing non-native aquatic species. Evidence indicates that 68 non-native aquatic species have escaped from aquaculture into natural habitats in China, successfully establishing wild populations, with 52 of these species posing risks to local aquatic ecosystems [12]. Shaanxi Province, as part of China’s northwest region, has a unique advantage in developing inland aquaculture. According to statistics from relevant departments, Shaanxi ranks first in the northwest region in terms of aquaculture area and freshwater aquaculture production [64]. For instance, *I. punctatus*, originally from North America, was introduced to China from Japan in the late 1970s and is primarily farmed in ponds in the upper Yellow River region. In the 1990s, *H. olidus* was introduced into the Liujiaxia Reservoir for large-scale farming, and surveys have shown that both *H. olidus* and *P. hyalocranius* have become dominant species in the area [65]. In the late 1980s, Shaanxi Province introduced *Rana catesbeiana* for special farming, mainly in the Guanzhong and southern Shaanxi regions. Due to market factors, many farmers abandoned *R. catesbeiana* farming, leading to the escape and spread of some *R. catesbeiana* and resulting in the establishment of natural populations [60]. The high invasion risks of many non-native aquatic animals in Shaanxi Province are largely due to the ongoing development of aquaculture practices. As an emerging industry, the ornamental trade has gradually become a major pathway for the introduction and establishment of fish [66].

### 4.3. The Barrier Role of the Qinling Mountains on Non-Native Aquatic Animals

Mountains cover a large part of the Earth’s surface and host unique ecosystems. Characterized by distinct landforms, high habitat heterogeneity, diverse climates, low human disturbance, and valuable ecosystem services, mountain regions play a crucial role in regulating species distribution and maintaining ecological balance [7,67]. The Qinling mountains range acts as a natural barrier between northern and southern China, shaping the biodiversity differences between these regions due to its unique terrain and climatic conditions [20]. Previous studies have indicated that among the most important factors for freshwater organisms are natural barriers, such as mountain ranges, which prevent the exchange of species among regions [29,68].

The number of non-native aquatic animals differs between the northern and southern sides of the Qinling Mountains. This pattern reflects the barrier function of the Qinling Mountains, operating through two complementary mechanisms that act at different stages of the invasion process. First, hydrological (physical) isolation limits cross-basin dispersal by reducing water connectivity, thereby constraining the arrival of propagules. Second, climatic differentiation between the two slopes acts as an environmental filter that affects the establishment and persistence of introduced populations once dispersal occurs. Accordingly, water connectivity plays a crucial role in dispersal in freshwater ecosystems [69]. Taking high-invasion-risk non-native aquatic animals, such as *Sander lucioperca*, as an example, habitat suitability predictions show that the region south of the Qinling Mountains is suitable for its survival. However, *S. lucioperca* has not been detected spreading south of the Qinling Mountains, likely due to the physical barrier effect of the mountains. This geographical barrier also directly affects the distribution of native species. For example, the northern slope of the Qinling Mountains has fewer fish species, with only 99 species, while the southern slope has a greater diversity, with 142 species [26]. The rapid uplift of the Qinling Mountains has distinctly divided China into northern and southern regions in terms of climate [70]. This uplift has led to warmer, wetter conditions in the south and cooler, drier conditions in the north. The Qinling Mountains block the influence of monsoons from the Indian and Pacific Oceans in the north, while preventing cold air from the north from reaching the south [20]. These climatic differences significantly affect the distribution of non-native aquatic species. Our research shows that non-native species in the north, such as *Hypomesus olidus*, *Protosalanx hyalocranius*, *Sander lucioperca*, and *Procambarus clarkii*, are typically adapted to lower temperatures or are temperature generalists. In contrast, species in the south, like *Carassius carassius var. germanus*, *Carassius carassius var. specularis*, *Megalobrama amblycephala*, and *Rana catesbeiana*, are adapted to warmer climates or are also generalists. This reflects the climatic gradient created by the mountains, where the mean annual temperature (Bio-01) is significantly lower in the north than in the south [71,72]. The Qinling Mountains act as a barrier, leading to significant temperature differences, particularly in winter. The average January temperature in the north is below 0 °C, while it exceeds 1 °C in the south. This temperature difference, which can be in the range of 6–7 °C, prevents cold air from the north from crossing into the south [73,74]. Our RDA analysis shows that Bio-01 is the primary factor influencing the invasion risk of high-risk non-native aquatic species. Species such as *S. lucioperca*, *P. hyalocranius*, and *H. olidus* are better suited to the cooler conditions north of the Qinling Mountains. For example, *H. olidus* has low habitat suitability in the south because the Bio-01 there exceeds its thermal tolerance for larval development, preventing the species from establishing populations [75]. This confirms that Bio-01 plays a critical role in shaping species distribution. Additionally, studies have shown a strong positive correlation between mean annual temperature and the richness of non-native species [28,76]. Our random forest model further supports this.

### 4.4. Influence of Human Activities on the Spread of Non-Native Aquatic Animals

The introduction of non-native species is a prominent issue associated with inter-basin water transfer, and aquatic invasions are becoming more frequent worldwide [77]. Inter-basin water transfers connect previously isolated watersheds, allowing aquatic organisms to disperse over long distances via artificial canals [78]. Inter-basin water transfer weakens the role of the Qinling Mountains as important geographical barriers. The water diversion from Hanjiang River to Weihe River runs through the Qinglin Mountains, which diverts water from the Yangtze River basin’s Hanjiang River to the Yellow River basin’s Weihe River, provides opportunities for cross-basin invasions by non-native aquatic animals. For example, the diversion of water through the East Route of the South-to-North Water Transfer Project (ESNT) has resulted in the invasion of the Shimofuri goby (*Tridentiger bifasciatus*), which has been detected in water storage lakes along the ESNT and has successfully established populations [79,80]. Additional, these transfers also modify environmental conditions, such as water physicochemical properties, hydrological patterns, and habitats, potentially disrupting recipient ecosystems and facilitating the establishment of introduced species [81,82]. For example, the Hanjiang-to-Weihe River project has created a series of reservoirs [83]. Studies have demonstrated that reservoirs are more susceptible to invasions than natural lakes, potentially due to the complex interplay of environmental factors unique to reservoir ecosystems [5]. Fluctuations in water levels driven by flood control and hydroelectric regulation disrupt native habitats by reducing large aquatic vegetation and degrading conditions suitable for native species, thereby promoting the establishment of invasive species [84]. Additionally, reservoirs create environmental conditions that favor the expansion of invasive and thermophilic species [85].

In addition to inter-basin water transfers, intentional human-mediated hybridization and selective breeding could potentially weaken the climatic filtering component of the Qinling Mountains’ barrier effect by increasing adaptive tolerance and facilitating establishment across climatic gradients [86,87]. Hybridization is considered an important source of adaptive allelic variation at the range edge, involving the interbreeding of distinct evolutionary groups or species [88,89,90]. The offspring of hybridization possess greater genetic diversity while also expanding the species’ range by transferring specific adaptive alleles [86]. For example, previous studies have shown that human activities have facilitated genetic mixing between different lineages of *Sander lucioperca* (invasive and native), bringing previously isolated genetic lineages into secondary contact. This has led to changes in genetic diversity, enhancing the adaptive potential of invasive populations [91]. Similar examples can also be found in the case of *Hypophthalmichthys molitrix*, where artificial hybridization led to the generation of multiple genotypes, accelerating the evolution of the species and enhancing the genetic capacity of *H. molitri* to adapt to new environments [92]. Moreover, in selective breeding, individuals that are robust (adaptable to a wide range of production environments or capable of maintaining internal balance) are often selected, allowing them to continue growing despite changes in the environment [87]. For example, selectively bred populations of *Cirrhinus mrigala* can naturally overwinter for over 50 days at temperatures below 10 °C, while all normal *C. mrigala* die under the same conditions, indicating a significant improvement in the cold resistance of the selectively bred *C. mrigala* [93]. Therefore, selectively bred species may adapt to harsh environments that the original populations could not, further weakening the impact of climate on changes in species distribution.

## 5. Conclusions

(1) Significant geographical barriers hinder the natural dispersal of non-native aquatic animals. Therefore, when monitoring, issuing early warnings, and managing non-native aquatic species in regions with geographical barriers, attention should focus on human-mediated pathways of cross-basin and cross-regional invasions. This is especially pertinent to human-engineered projects that overcome geographical barriers, such as the “Hanjiang River to Weihe River water diversion” project. In addition, it is important to remain aware of the risks posed by hybridization and selective breeding of species resulting from human activities, as these could potentially break traditional species distribution boundaries and lead to more extensive invasions. (2) Allocate monitoring resources for non-native aquatic animals based on scientific predictions of potential suitable habitats for high-risk species. This strategy will assist in identifying priority monitoring areas and establishing fixed monitoring sites for long-term tracking of species distribution and population dynamics. In areas with lower invasion risks, periodic mobile monitoring should be employed to improve overall monitoring efficiency.

## Figures and Tables

**Table 1:** Description of environment variables.

Environment Variable	Variable Description	Units
Bio-01	Annual Mean Temperature	°C (×10)
Bio-02	Mean Diurnal Range	°C (×10)
Bio-03	Isothermality	% (×100)
Bio-04	Temperature Seasonality	°C (×100)
Bio-05	Max Temperature of Warmest Month	°C (×10)
Bio-06	Min Temperature of Coldest Month	°C (×10)
Bio-07	Temperature Annual Range	°C (×10)
Bio-08	Mean Temperature of Wettest Quarter	°C (×10)
Bio-09	Mean Temperature of Driest Quarter	°C (×10)
Bio-10	Mean Temperature of Warmest Quarter	°C (×10)
Bio-11	Mean Temperature of Coldest Quarter	°C (×10)
Bio-12	Annual Precipitation	mm
Bio-13	Precipitation of Wettest Month	mm
Bio-14	Precipitation of Driest Month	mm
Bio-15	Precipitation Seasonality	1
Bio-16	Precipitation of Wettest Quarter	mm
Bio-17	Precipitation of Driest Quarter	mm
Bio-18	Precipitation of Warmest Quarter	mm
Bio-19	Precipitation of Coldest Quarter	mm
Elevation	Elevation	m

**Table 2:** Catalog of non-native aquatic species and distribution in the Qinling Mountains (narrow sense). Note: AB, abroad; ORC, other regions of China; DSB, different sections of the same river basin.

Species/Variety	AB	ORC	DSB	Basin Distribution	Longitude	Latitude	Introduction Methods	Native/ Non-Native	Data Source
*Cyprinus carpio* *var. germanus*	+			YRB	110.4896583	36.46724986	Aquaculture	Non-native	Field Collection
				YZRB	108.8391726	32.59056207		Non-native	[51]
*Cyprinus carpio Red* * var wuyuanensis*		+		YRB	110.2821992	34.60939743	Ornamentals Trade	Non-native	[52]
*Cyprinus carpio* * var. specularis*	+			YRB	111.0610978	39.02323666	Aquaculture	Non-native	[53]
				YZRB	108.8686632	32.57220189		Non-native	[54]
*Tinca tinca*		+		YZRB	107.536985	33.590466	Aquaculture	Non-native	Field Collection
					108.2393799	33.13554845		Non-native	[55]
*Carassius auratus*		+		YRB	110.6098642	34.60986417	Aquaculture	Non-native	[53]
*Carassius auratus gibelio*		*+*		YRB	110.6098642	34.60986417	Aquaculture	Non-native	[53]
					110.3640201	35.14246161		Non-native	
					111.0604552	39.02374077		Non-native	
*Megalobrama amblycephala*		+		YRB	110.6098642	34.60986417	Aquaculture	Non-native	[56]
*Hemibarbus umbrifer*		+		YRB	110.6098642	34.60986417	Unintentional transport	Non-native	[53]
*Luciobarbus capito*	*+*			YRB	110.9072255	34.6897459	Aquaculture	Non-native	Field Collection
					110.6098642	34.60986417		Non-native	[53]
*Rhodeus fangi*		*+*		YRB	110.6098642	34.60986417	Unintentional transport	Non-native	[57]
				YZRB	107.1486177	33.08442392		Non-native	[54]
*Myxocyprinus asiaticus*		+		YRB	110.6098642	34.60986417	Ornamentals Trade	Non-native	[57]
*Clarias gariepinus*	+			YRB	110.6317709	37.39504955	Aquaculture	Non-native	Field Collection
*Silurus meridionalis*		+		YRB	111.0604552	39.02374077	Aquaculture	Non-native	[53]
*Ictalurus punctatus*	*+*			YRB	110.4975048	35.356952	Aquaculture	Non-native	Field Collection
				YZRB	107.0286975	33.05141128		Non-native	[58]
*Oreochromis niloticus*	+			YRB	110.3640201	35.14246161	Aquaculture	Non-native	[53]
*Sander lucioperca*	+			YRB	111.4154075	39.49586436	Aquaculture	Non-native	Field Collection
*Hypomesus olidus*		+		YRB	110.6335853	37.38954325	Aquaculture	Non-native	Field Collection
*Protosalanx hyalocranius*		*+*		YRB	110.1702808	34.63822908	Aquaculture	Non-native	[59]
				YZRB	110.1191199	32.81353236		Non-native	[55]
*Acipenser gueldenstaedti*	*+*			YRB	110.6098642	34.60986417	Aquaculture	Non-native	[53]
					110.3640201	35.14246161		Non-native	
					111.0604552	39.02374077		Non-native	
*Hemiculterella sauvagei*		+		YZRB	108.8391726	32.59056207	Stocking for Fisheries	Non-native	[54]
*Spinibarbus denticulatus*		+		YZRB	108.8391726	32.59056207	Aquaculture	Non-native	[54]
*Procambarus clarkii*	*+*			YRB	110.6098642	34.60986417	Aquaculture	Non-native	[53]
					110.3640201	35.14246161		Non-native	
				YZRB	107.0520549	33.04479526		Non-native	[60]
*Eriocheir sinensis*		*+*		YRB	110.6098642	34.60986417	Aquaculture	Non-native	[53]
					110.3640201	35.14246161	Stocking for Fisheries	Non-native	
					111.0604552	39.02374077		Non-native	
*Chelydra serpentina*	*+*			YRB	110.3640201	35.14246161	Ornamentals Trade	Non-native	[53]
					108.7187896	34.32788695		Non-native	[61]
*Trachemys scripta elegans*	*+*			YRB	108.7187896	34.32788695	Aquaculture	Non-native	[61]
					107.1593223	34.36127724		Non-native	
					109.0168808	34.4316423		Non-native	
				YZRB	109.0466687	32.70392889	Ornamentals Trade	Non-native	[60]
*Trionyx Sinensis*			*+*	YRB	110.6335853	37.38954325	Aquaculture	Non-native	Field Collection
				YRB	108.7187896	34.32788695	Stocking for Fisheries	Non-native	[61]
*Rana catesbeiana*	+			YZRB	107.0183112	33.04927819	Aquaculture	Non-native	[53]

**Table 3:** Distribution of non-native aquatic animals across different basins within the Qinling Mountains (narrow sense).

Watershed	Mainstream	Tributaries	Number
Yangtze River Basin		Hanjiang River	11
		Jialingjiang River	0
			0
Yellow River Basin	Mainstream of the Yellow River		23
		Weihe River	5

**Table 4:** Invasion risk assessment scores of non-native aquatic animals in Qinling Mountains. Note: IS, invasion history (1 = yes; 0 = no); BRA, Basic Risk Assessment score; CCA, Climate Change Assessment score; BRA + CCA, combined risk score; Level, invasion risk level based on the corresponding score.

Species/Variety		Evaluate Results				Confidence		
	IS	BRA	Level	BRA + CCA	Level	BRA + CCA	BRA	CCA
*Trachemys scripta elegans*	1	40	High	52	High	0.5	0.49	0.63
*Procambarus clarkii*	1	36.5	High	48.5	High	0.54	0.54	0.5
*Rana catesbeiana*	1	35.5	High	47.5	High	0.54	0.51	0.83
*Tinca tinca*	1	34	High	46	High	0.52	0.51	0.63
*Chelydra serpentina*	1	33.5	High	45.5	High	0.55	0.53	0.67
*Sander lucioperca*	1	35	High	43	High	0.58	0.59	0.5
*Protosalanx hyalocranius*	1	36	High	42	High	0.52	0.51	0.63
*Hypomesus olidus*	1	35	High	41	High	0.51	0.5	0.58
*Megalobrama amblycephala*	1	31.5	High	37.5	High	0.54	0.54	0.54
*Ictalurus punctatus*	1	30.5	High	36.5	High	0.53	0.53	0.5
*Cyprinus carpio var. germanus*	1	26	Medium	34	High	0.63	0.62	0.67
*Cyprinus carpio var. specularis*	1	25.5	Medium	33.5	High	0.62	0.62	0.67
*Carassius auratus gibelio*	1	27	High	33	High	0.53	0.51	0.71
*Silurus soldatovi meridionalis*	1	12	Medium	10	Medium	0.61	0.6	0.71
*Oreochromis niloticus*	1	−1	Low	−7	Low	0.85	0.88	0.63
*Clarias gariepinus*	1	−2	Low	−8	Low	0.9	0.88	1
*Luciobarbus capito*	0	26.5	Medium	32.5	Medium	0.61	0.63	0.46
*Carassius auratus*	0	25	Medium	27	Medium	0.57	0.56	0.67
*Cyprinus carpio Red var wuyuanensis*	0	15	Medium	21	Medium	0.61	0.59	0.83
*Acipenser gueldenstaedti*	0	18	Medium	18	Medium	0.53	0.5	0.79
*Trionyx Sinensis*	0	−2	Low	−2	Low	0.92	1	0.25
*Eriocheir sinensis*	0	−2	Low	−2	Low	0.92	0.93	0.88
*Myxocyprinus asiaticus*	0	−3	Low	−9	Low	0.92	0.92	0.88
*Hemiculterella sauvagei*	0	−4	Low	−6	Low	0.54	0.54	0.58
*Spinibarbus denticulatus*	0	−5	Low	−5	Low	0.57	0.56	0.63
*Rhodeus fangi*	0	−8	Low	−12	Low	0.92	0.94	0.75
*Hemibarbus umbrifer*	0	−7	Low	−13	Low	0.91	0.9	1

## Data Availability

Data will be made available on request.

## Supplementary Materials

The following supporting information can be downloaded at: https://www.mdpi.com/article/10.3390/biology15040329/s1, Table S1: Model performance (AUC) for MaxEnt predictions and numeric ranges for habitat suitability classes (Figure 2); Table S2: Environmental variables used in the redundancy analysis (RDA); Table S3: Species number, fisheries output value, and aquatic product yield on the northern and southern sides of the Qinling Mountains.
